# A network-based analysis of bacterial growth on substrate mixtures uncovers glucose inhibition in *Phaeobacter inhibens*

**DOI:** 10.1038/s41598-025-33583-6

**Published:** 2026-01-08

**Authors:** Leonhard Lücken, Bernd Blasius

**Affiliations:** 1 Institute for Chemistry and Biology of the Marine Environment (ICBM), Carl von Ossietzky Universität Oldenburg, Oldenburg, Germany; 2 Helmholtz Institute for Functional Marine Biodiversity, Oldenburg, Germany

**Keywords:** Bacterial growth, Substrate mixtures, Inhibition network, Substitutable resources, Carbon catabolite repression, Diauxie, Microbial ecology, Regulatory networks, Marine microbiology, Bacterial systems biology, Network topology

## Abstract

Many bacteria exhibit the remarkable ability to thrive on a diverse range of nutritional substrates. This allows them to survive in environments with a complex composition of resources, even if their total availability is low. The optimal regulation of resource usage is crucial for the ecological success in such environments. Thus, understanding its regulatory facilities can help to understand the ecological function of a microbial species. However, in a complex regulatory context, changing substrate preferences can be challenging to detect from limited experimental data without a systematic analysis. In this work, our primary objective is to introduce a method to infer potential substrate interaction networks in single-species heterotrophic bacterial cultures growing on mixed substrates. Our approach compares substrate depletion curves and can reveal the most important pairwise interdependencies between uptake and presence of substrates. We applied our method to previously published batch culture experiments with the marine bacterium *Phaeobacter inhibens*, where a mixture of sugars and amino acids served as the exclusive carbon source. This analysis revealed a coexistence of diauxie and co-utilization of substrates illustrating complex regulatory strategies in this bacterium. Notably, we found an inhibition of glucose uptake by the availability of other sugars, i.e., mannitol and/or N-acetylglucosamine. This contrasts the well-documented prioritization of glucose in many other bacteria.

## Introduction

Over the last decade, biologists directed an increasing effort to the study of marine microbiota, unveiling the existence of complex regulatory interaction networks within microbial ecosystems that populate our planet’s oceans^1^. Especially the important role of microorganisms in biogeochemical cycles has emphasized the need for a deeper understanding of the associated dynamics^2^. Given the substantial fluctuations in nutrient availability that marine ecosystems often experience, many organisms have evolved versatile metabolic capabilities for exploiting these ever-changing conditions effectively, including the strategic formation of reserves^3^ and the adoption of dietary flexibility^4^. Depending on the prevailing nutrient conditions, two primary nutritional modes emerge as fundamental survival strategies: In environments characterized by a low availability of organic carbon substrates microorganisms adopt a strategy of simultaneously consuming a wide variety of substrates^4,5^. Conversely, in environments where carbon sources are abundant, where other factors, e.g., inorganic nutrients, are limiting, organisms generally exhibit sequential utilization of compounds, often resulting in “diauxic growth,” where the preferred substrate is consumed first^6–8^.

A widely used approach for studying microbial resource usage strategies is through laboratory experiments. In batch cultivation, where substrates are not replenished, the utilization of substrates in a sequential manner gives rise to auxic shifts, which manifest in distinct growth phases, a phenomenon that was recognized more than 75 years ago by Jacques Monod^9^. The transition between these phases may or may not involve auxic lags corresponding to the underlying processes of metabolic adaptation within the population. While this phenomenon has primarily been studied in laboratory settings where two substitutable substrates are provided, more complex relationships can occur in environments with greater substrate diversity. This can result in tri-, or multi-auxic shifts, as well as the simultaneous co-utilization of multiple substrates^10,11^.

Typically, the most preferred compounds are those that allow for highest growth rates^4,12^, although this rule is not without exception^13,14^. Corresponding adaptive heterotrophic strategies have been theoretically derived in models based on principles optimizing growth success under varying substrate compositions. A relatively early class of models, which are able to reproduce sequential and simultaneous resource usage are “cybernetic models”, which successfully explained regulatory strategies in terms of optimal resource allocation, but without including a detailed account of metabolic pathways^15^. More recent advances in understanding the molecular mechanisms underlying substrate preference have led to the development of various modeling approaches to describe diauxic shifts. One such approach is dynamic flux balance analysis (dFBA) of metabolic networks^16–18^ which was applied successfully to explain variable substrate preferences as a consequence of optimizing specific variables (e.g., growth rate or ATP production). Predictions of microbial usage patterns have been further improved by complementing dFBA models with resource allocation constraints^19^. Further, substantial progress has been made in the last decade in the detailed mechanistic modelling of regulatory mechanisms involved in diauxic shifts^20^. These advances have significantly improved our understanding of the mechanisms underlying auxic shifts, but they require extensive computational resources, detailed kinetic parameters, or organism-specific, curated metabolic networks. Detailed information is typically only available for model organisms, constraining the applicability of such approaches for less-studied species.

Here, our main goal is to present a methodological framework for inferring potential substrate interactions from growth data (substrate concentration and population density time series) in single-species microbial cultures. The approach uses a relatively small set of parameters and requires no organism-specific information, making it broadly applicable across diverse microorganisms. It identifies candidate pairwise inhibitory relationships between substrates, but it cannot resolve their mechanistic basis (e.g., enzymatic competition, regulatory repression, or byproduct inhibition). Thus, further experimental validation is required to elucidate the hypothesized interactions. Further, to demonstrate the method’s utility, we apply it to the heterotrophic marine bacterium *Phaeobacter inhibens *grown on a mixture of eleven carbon sources^21^.

*P. inhibens*is a member of the Roseobacter group, which often dominates bacterioplankton during phytoplankton blooms^22^. Its ecological success reflects adaptations to fluctuating resources, where dynamic substrate utilization strategies are critical. Unlike model organisms like *E. coli* that enforce strict sequential uptake (diauxie) under abundant substrate availabilities, *P. inhibens* regularly displays strategies including simultaneous co-utilization of substrates. Notably, Zech et al.^23^ observed that in complex media (containing mainly amino acids and phospholipids), *P. inhibens* exhibited a pronounced downregulation of glucose metabolism. In another study, whose experimental results we revisit in this study, Wünsch et al.^21^ reported rate-regulated hierarchical depletion in defined mixtures, where sugars are consumed faster than amino acids. Both studies emphasize the metabolic versatility of *P. inhibens* and align with complex substrate interaction strategies in other marine heterotrophs, such as the sequential co-utilization of amino acid clusters in *Pseudoalteromonas haloplanktis*^11^. Its demonstrated capacity for diverse regulatory responses to substrate combinations makes *P. inhibens* a promising model for studying substrate interaction networks.

For growth simulations we use a conceptual dynamic energy budget (DEB) model [Eq. (1)]. DEB models are a class of mechanistic models that describe how organisms allocate energy for growth, maintenance, and sometimes reproduction, based on energy intake from substrates^24^. Unlike constraint-based approaches like dFBA, DEB models use simplified physiological rules to capture growth dynamics, thereby remaining computationally feasible for exploring complex substrate interactions. While DEB theory has originally been developed to describe the growth of an individual organism, it has been adapted to a variety of different settings ranging over all organism sizes from bacteria to animals and over modeling scales of sub-cellular to population dynamics^25,26^. In particular, DEB population models have been successfully applied to model populations of marine heterotrophic bacteria^3^. Although the protocol for incremental identification of substrate interaction proposed below does not depend crucially on the phenomenological model adopted for its implementation, the DEB approach is most suitable for the case of *P. inhibens* since it allows us to incorporate known substrate energy yields^21^.

In our model, we include the general mechanism that determines the occurrence of auxic shifts and co-utilization by an inhibitory regulatory network between substrates, such that the presence of one or more substrates inhibits the utilization of other substrates to varying degrees. Although the molecular details of these inhibitory interactions may take different forms in reality, such as enzyme synthesis repression or allosteric inhibition, as a first order of approximation a coarse-grained model can assume a common effective form for each inhibitory relationship^12^. This approach becomes particularly valuable when attempting to detect regulatory relationships in a complex situation where the presence and specific implementation of these relationships are unknown. The interdependence in the utilization of different substrates can then be captured in a model by incorporating an uptake rate for each substrate, which is adjusted dynamically based on the availability of other substrates. Several studies have presented such models to describe the simultaneous or concurrent consumption of multiple substrates^10,12,27,28^. However, while these models hold promise for addressing more complex substrate compositions, their practical validation typically focuses on mixtures containing only two different substrates.

In the “Methods” section, we lay out a generic procedure to generate hypotheses about inhibitory relationships between substrates in growth experiments on complex substrate mixtures. The procedure systematically assesses, which of the possible relationships exhibits the most significant explanatory benefit to arrive at a hypothesis concerning the presence of regulative substrate interactions. We employ this approach to analyze the growth of *P. inhibens *on a mixture of eleven substrates (three sugars and eight amino acids)^21^. Our model for *P. inhibens *uses a general form of substrate interaction, originally proposed by Yoon et al.^12^, which can arise from generalized binding kinetics of sequentially processed substitutable substrates^28^. Importantly, our method is not dependent on a specific mathematical model, as alternative variants of growth dynamics can be incorporated provided they parameterize substrate-dependent inhibition.

## Results

### Detection of substrate interactions for *P. inhibens*

**Fig. 1:**
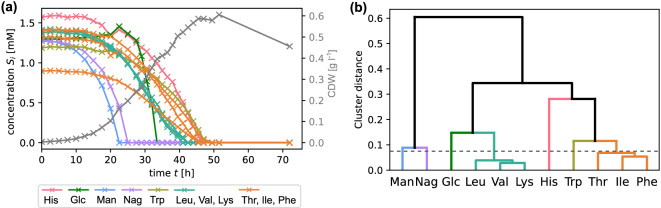
Substrate clustering based on an experiment with the marine bacterium *P. inhibens*, growing on multiple substrates. (**a**) Time series for one experimental replicate, showing the temporal course of substrate concentrations, colored according to their cluster association as indicated in the legend, and the cell dry weight (gray curve). See section “Experimental data” for substrate abbreviations. (**b**) Hierarchical clustering of substrates obtained from the distance (4) [see section “Methods”] using farthest point cluster distances. The dashed line indicates a cut height of 0.075.

We analyzed inhibitory relationships between substrates in a culture of the marine bacterium *P. inhibens* originally conducted by Wünsch et al.^21^. Figure 1(a) shows the depletion curves of the different substrates and the population growth indicated by cell dry weight (CDW) measurements during the experiment. Most notably, although all substrate concentrations declined monotonically, *P. inhibens* exhibited distinct substrate-specific utilization patterns. For example, mannitol and N-acetylglucosamine were were depleted much faster than the other substrates. Before we begin to analyze the mechanistic reason for this, we note a fundamental limitation of identifying inhibitory relationships from a single experiment: In the case that the presences of two potentially inhibitory substrates coincide very closely, both could equally well explain an inhibition. In other words, depletion curve similarities correspond to a lack of parameter identifiability^29^. Such a situation can imply technical difficulties for model selection. Therefore, as a prerequisite to further analysis, we clustered substrates into classes exhibiting similar depletion curves [Fig. 1(b)].

To explain the growth curves, obtained in the experiment, we developed a conceptual DEB model describing the growth dynamics of *P. inhibens* on a mixture of carbon sources, with inhibitory effects between the presence of one and the uptake of another substrate [Eq. (1)]. For details, we refer to the section “Bacterial growth model” under “Methods”. To determine the influence of other substrates on a substrate *i*, we repeatedly fitted the uptake part of the model [Eq. (5)] assuming the presence of up to two different interactions. By separately fitting only the uptake dynamics, the set of simultaneously optimized parameters is greatly reduced in comparison to the full model. Indeed, Eq. (5) has only two basic parameters (the maximal uptake rate $\mu _i$ and the half-saturation constant $K_i$), and an additional coefficient $a_{i,j}$ for each included interaction. This decoupled fitting enhances the computational feasibility of the problem and allows a broad scanning of possible combinations of inhibitory effects.

**Fig. 2:**
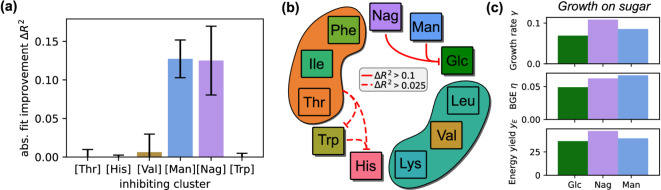
(**a**) Fit improvement $\Delta R^2$ obtained by including different inhibitors for glucose. Error bars indicate standard deviations across resampled fits. (**b**) Inhibitory network derived from error reductions by including one inhibitor. Interactions, which yield a fit improvement $\Delta R^2>0.1$ with respect to the model without interactions are drawn as solid red lines, while interactions with $0.1>\Delta R^2>0.025$ are indicated by dashed red lines. (**c**) Growth characteristics for *P. inhibens* cultures on a single sugar.

Based on the comparison of different model fits, we calculated the improvements induced by including an additional interaction for all substrates (see “Methods” section). For *P. inhibens*, significant improvements were observed for only a small subset of substrates, *cf.* Supporting Information (SI), Fig. S2(b). This improvement was by far the largest for the glucose fit, where the uptake appears to be strongly inhibited by the presence of either N-acetylglucosamine ($R^{2} = 0.94$) or mannitol ($R^{2} = 0.91$), *cf.* SI Fig. S2(a). As shown in Fig. 2(a), this yields a fit improvement of $\Delta R^2>0.1$ ($\Delta R^2_{Man} = 0.13$ and $\Delta R^2_{Nag}=0.15$) with respect to the glucose fit without interactions ($R^{2} = 0.79$). Considering other substrates as potential inhibitors did not improve the glucose fit significantly. We have repeated the fitting protocol using subsets of the experimental replicates, i.e., all combinations of $\ge 2$ of the four replicates. The standard deviations of $\Delta R^2$ obtained across these combination for glucose inhibition are shown as error bars in Fig. 2(a).

A network of potential substrate interactions can be stipulated based on the different improvements of fits (or the relative reduction of errors, *cf.* SI Section S2.1). Networks obtained from significance thresholds $\Delta R^2>0.1$ (solid links) and $\Delta R^2>0.025$ (additional dashed links) are visualized in Fig. 2(b). It appears that the inhibition network in the substrate usage of *P. inhibens* is sparse at least for the tested mixture of sugars and amino acids, with only one or two strong interactions (inhibition of glucose uptake by N-acetylglucosamine and/or mannitol) and potentially two or three weak interactions.

**Fig. 3:**
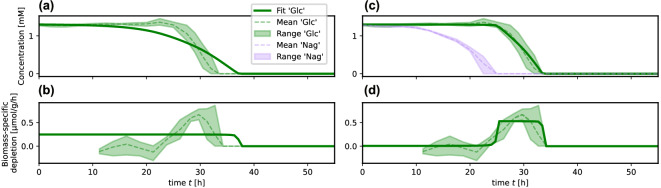
Model fits of system (5) for glucose depletion. Solid green curves show simulated concentrations (a;c) and depletion rates (b;d) of glucose; Shaded areas indicate observed ranges across all experimental replicates; dashed curves show mean values across all replicates. (**a**) and (**b**): Simulation without uptake inhibition by other substrates ($\mu = 0.25$, $K = 0.03$, $R^{2} = 0.79$); (**c**) and (**d**): inhibition by N-acetylglucosamine ($\mu = 0.53$, $K = 3\cdot 10^{-3}$, $a_{Glc,Nag} = 110.0$, $R^{2} = 0.94$).

In the case of the glucose uptake inhibition, we find that both optimal fits (for mannitol and N-acetylglucosamine) yield strong interaction coefficients ($a_{Glc,Nag} = 110.0$ for the simultaneous fitting to all experimental replicates and a standard deviation 175.6 over all subsets of two or more replicates, resp. $a_{Glc,Man} = 454.0$ with standard deviation 277.0), effectively blocking significant glucose uptake (specific uptake rate <0.1/h) while detectable concentrations of inhibitory sugars persist ($>50 \upmu$M)^21^. In this way, the model with interaction is able to reproduce the sequential usage of sugars as observed in the experiments. Figure 3 shows the fitted model for glucose uptake without inhibition [(a) and (b)], and with inhibition by N-acetylglucosamine [(c) and (d)].

### Comparison of cultures grown on mixed and individual carbon sources

Wünsch et al.^21^ also cultured *P. inhibens* on individual substrates [data shown in SI Fig. S5]. To identify systematic relationships between substrate quality and altered uptake characteristics for growth on the mixture, we calculated three growth performance indicators for all substrates [*cf.* Fig. 2(c) and SI Sec. S3]:

- The directly fitted, effective growth rate $\gamma$,
- Bacterial growth efficiency (BGE) $\eta$ during the exponential growth phase,
- ATP yield $y_E$ as computed theoretically by Wünsch et al. (SI Table S1).

For all indicators, glucose exhibits the least optimal values. Mannitol allows the highest growth efficiency, while for N-acetylglucosamine *P. inhibens* displayed the highest growth rate and the ATP yield per catabolized molecule $y_E$is highest. Since glucose seems to be the inferior substrate, its observed inhibition in presence of all sugars is in line with the idea of a growth-optimizing regulation^18^. The uptake of amino acids is much less selective, even though several (notably lysine, valine, and histidine) consistently showed inferior growth performance across all indicators ($\gamma$, $\eta$, $y_E$) compared to other amino acids (SI Fig. S5). Instead, we observe a simultaneous uptake with a significant reduction of individual uptake rates for all amino acids when comparing cultures on individual substrates to cultures on mixtures. The magnitude of the reduced uptake rates shows no clear relationship to the substrate quality indicators derived from growth on individual amino acids.

### Full model fit

**Fig. 4:**
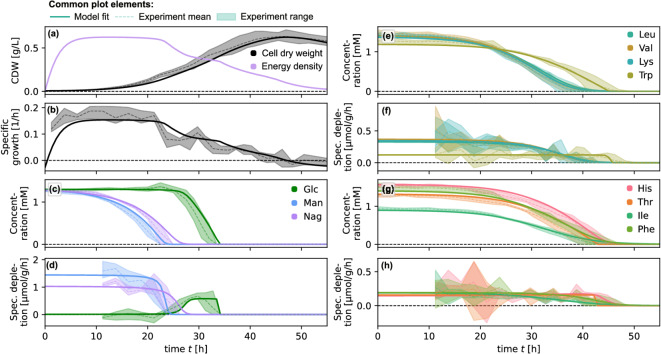
Dynamics of the full *P. inhibens* model fit with inhibition of glucose by N-acetylglucosamine (strong interaction) and histidine by threonine, isoleucine, and phenylalanine (weak interaction). Common elements: Solid curves show model output, dashed curves show mean values for observation points across all four experimental replicates, shaded areas indicate corresponding ranges. (**a**) Measured cell dry weight and biomass-specific energy reserve; (**b**) biomass-specific growth rate; (**c**)–(**h**) substrate time series are distributed over several panels to enhance visibility.

Figure 4 shows trajectories of a fit of the full model (1), that is, a simultaneous optimization of all model parameters with respect to a corresponding square error obtained from summing up the substrate errors [Eq. (6)] and the biomass error [see SI Eq. (S4)]. The number of parameters for the full system is 31 for the considered case: 2 parameters for each of the 11 substrates, 2 interactions, 3 growth parameters and 4 initial biomass concentrations (one for each experimental replicate). To successfully fit all parameters simultaneously, it was crucial to provide the optimization routine (a modified Powell algorithm implemented in SciPy’s^30^ minimize() method) initial parameter values according to the parameters obtained from the previous decoupled uptake model fits [and from a separate fit of the growth model – *cf.* SI Eq. (S3)]. For the full model, we only included the inhibition of glucose by N-acetylglucosamine and of histidine by the cluster consisting of threonine, isoleucine, and phenylalanine.

The model was only slightly improved by re-fitting the parameters for the full system. We obtained $R^2 = 0.98$ for the refitted parameters and $R^2 = 0.91$ for the full model run with the parameter values equal to the decoupled fits. Without any interactions, the full model was still able to explain a fraction of variance as high as $R^2 = 0.97$, such that the fit improvement of the full model with interactions with respect to the one without interaction is merely $\Delta R^2 = 7\cdot 10^{-4}$. This is a much weaker indication for the benefit of including interactions than in the case of individual decoupled fits. Thus, even if a full fit was possible without decoupled pre-fitting, in view of the reduced computational effort and the clarity of indication, the latter seems advisable for determining interactions.

## Discussion

Cases of catabolite repression, where glucose acts as the inhibitor are much better known and probably more widespread than cases where it is inhibited^31,32^. However, prioritization of glucose is not without known exception. For instance, Parche et al.^33^ report examples of glucose uptake inhibition by lactose. This is the exact opposite of the best-known example of catabolite repression, namely lactose inhibition by glucose in *E. coli*. If mannitol is the inhibitor in the case of *P. inhibens*, that would likewise stand in contrast to the reverse inhibition of mannitol by glucose in *E. coli*^34^.

Using dFBA, the sequential usage of carbon sources can often be explained as the result of metabolic optimization^16,17,19^. Regarding the sequential consumption of sugars found for *P. inhibens*, we showed that, when supplied exclusively, N-acetylglucosamine and mannitol permit higher growth rates, more efficient growth, and higher ATP yields than glucose [Fig. 2(c)]. This suggests that *P. inhibens *prioritizes substrate uptake to maximize growth rate and efficiency during transient high-carbon conditions like phytoplankton blooms, where substrate concentrations reach mM levels. Under such elevated concentrations, regulatory hierarchies favoring sequential utilization are well-documented^4–6^. Conversely, at oligotrophic concentrations (up to a few $\mu$M per substrate), simultaneous substrate co-utilization predominates^5,7^. Notably, the experiments analyzed here reveal that simultaneous utilization can also occur under high substrate availability, as *P. inhibens *continuously consumed all amino acids (initially 1.3 mM each) without diauxic shifts or inhibition patterns. This coexistence of diauxie and co-utilization has only recently been reported for another marine heterotrophic bacterium^11^. For *P. inhibens*, the comparison of correlations between substrate quality and fitted uptake parameters (for exclusive vs. simultaneous availability) of amino acids remained inconclusive (SI Fig. S6). A clearer principle governing the modified uptake rates for the growth on mixtures might be revealed by a more detailed metabolic model, which should also encompass anabolic pathways.

In this context it is worth noting that seemingly non-optimal behavior of specific bacteria have been reported by several studies^13,14,35^. Subsequent conceptual modeling studies have shown that such strategies can mitigate direct competition between community members and yield advantages for individual species. For instance, Wang et al.^36^ suggested to explain the emergence of complementary resource preferences by a community assembly model for diauxically growing populations, and Pacciani-Mori et al.^37^ studied a model describing the co-evolution (resp. co-adaptation) of microbial consumers, and observed co-existence owing to different resource utilization strategies. The interaction networks inferred by our method – such as the preferential use of mannitol and N-acetylglucosamine over glucose in *P. inhibens* – provide testable candidates ecological adaptations. While growth optimization likely drives this hierarchy for *P. inhibens*, identified relationships invite investigation into whether they also confer other advantages in natural communities (e.g., niche separation during algal blooms) via co-culture experiments or environmental metatranscriptomics.

While our approach identified candidate interactions (glucose inhibition) for *P. inhibens*, it has limitations that should be noted. First, the model defined by Equation (1) assumes that metabolic responses essentially take the form of inhibition relief and take effect immediately. The very close fits ($R^{2} \approx 0.94$) of the simulated experiment suggest that this assumption captures the most important responses for the *P. inhibens* cultures on mixtures, but it may not generalize. This is evidenced by the failure of predicting the growth on the mixture by uptake parameters fitted to single-substrate growth (SI Sec. S3), suggesting slower acclimation processes adapting the metabolic configuration of cultures to different nutritional environments. Second, identifiability challenges arise when depletion curves exhibit similarity, obscuring the specific inhibitor responsible for an effect (e.g., distinguishing whether mannitol or N- acetylglucosamine inhibits glucose). Such ambiguities could be targeted follow-up experiments, such as pairwise cultures (e.g., glucose-mannitol vs. glucose-N-acetylglucosamine), implying a top-down workflow where candidate interactions from complex mixtures are validated in reduced subsets. Third, noise and scalability limitations emerged in surrogate testing (SI Sec. S4), where the accuracy of our method declined with increasing interactions or measurement noise. Despite these limitations, our approach offers distinct advantages for the exploratory screening of substrate interactions in complex environments. Unlike dFBA, which requires *a priori* knowledge of genome-scale metabolism, or machine learning methods that demand large training datasets, our model efficiently identifies candidate interactions from limited experimental data (i.e., time-series of population density and substrate concentrations). This parsimony makes our method useful for initial investigations of microbial systems where regulatory mechanisms are unknown or metabolic models are unavailable – as demonstrated by its successful application to *P. inhibens*.

Substrate usage in complex mixtures is highly relevant to microbial ecology, environmental and human microbiome research, as well as microbial biotechnology. Given the considerable effort required for growth experiments on complex compound mixtures, it is important to develop methods to extract as much information as possible from this valuable data. Analytic tools for an exploratory identification of possible regulative relationships represent an initial step into that direction and our approach provides multiple avenues for future research in this context: From an applied perspective, future work should test the approach in dynamic substrate regimes relevant to biotechnological processes or environmental scenarios, such as fed-batch cultures with controlled substrate influx, systems simulating abrupt nutrient transitions (e.g., substrate pulses or depletion-replenishment cycles), or sequential substrate addition regimes mimicking ecological succession dynamics. Highly relevant applied scenarios also include multi-species systems, which our current framework doesn’t address, currently. A future extension could screen species interactions using a similar approach as we devised for substrate interactions. A fundamental question is whether inhibition networks are typically sparse (as observed here) or exhibit more complex regulatory patterns – a distinction with important consequences for our understanding of microbial growth dynamics in natural environments, though more experimental data is needed to explore this. Methodologically, advancements could incorporate alternative growth kinetics, such as adaptive metabolism, and explicit metabolite production costs; evaluate extended functional forms such as higher Hill coefficients for inhibition; and integrate community dynamics. Further, future work could implement more robust confidence analysis for parameter estimation and interaction impact evaluation (e.g., via likelihood profiles or bootstrapping), though computational cost has to be considered^38^. For *P. inhibens*, important biological characteristics include its complex post-exponential behavior – notably cell shape shifts, biofilm formation, and conglomeration – and the roles of exometabolites. While tropodithietic acid (TDA) production costs were subsumed into maintenance due to their lack of correlation with substrate usage here, TDA’s antibiotic or quorum-sensing functions may link to metabolic regulation in other scenarios.

## Methods

### Bacterial growth model

In this work, we employed a conceptual DEB model to describe the population dynamics of a microbial consumer species, and the associated consumption of substrates in a batch reactor. Key features of DEB models are the explicit representation of an energy reserve and maintenance costs for structural biomass^24^. We followed Kooi and Kooijman^39^, who derived an unstructured population model for *E. coli *based on approximating the individual bacterial cell as a V1-morph, i.e., as having a cell surface proportional to its volume (e.g. growing as filaments or rods). More elaborated structured population models based on DEB principles have been proposed, which describe the heterogeneous energy reserve and biomass distribution of a population^26^.

**Fig. 5:**
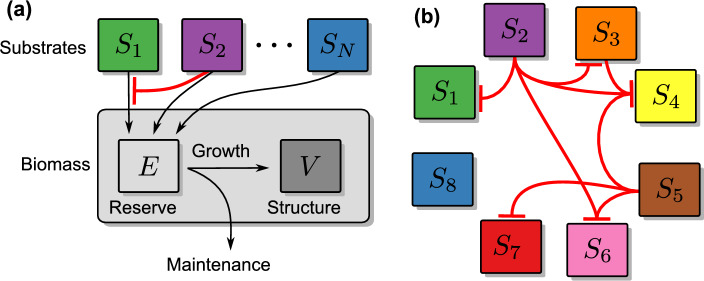
Overview of the model (1); (**a**) *N* different substrates $S_{1},...,S_{N}$ are assimilated into an internal energy reserve *E*, which is used for building up and maintaining the structural biomass *V* of the population. The red, blunt-head line represents an exemplary inhibitory effect of the presence of $S_{2}$ on the uptake of $S_{1}$, i.e., $a_{1,2}>0$ in Eq. (2). (**b**) A hypothetical network of inhibitory effects $a_{i,j}\ne 0$.

The population model used in the following incorporates a total energy reserve *E*, which is maintained by the uptake of a variety of *N* different substrates $S_{i}$, $i=1,...,N$. This reserve is then used for expenditures proportionally allocated for cell maintenance and the growth of structural biomass *V* (see schematic overview in Fig. 5(a)). The temporal evolution of the system’s state is assumed to be governed by the following set of differential equations:

1 $$\begin{aligned} \dot{S}_{i}&=-f_{i}\left( S_{1},...,S_{N}\right) \cdot V,\nonumber \\ \dot{E}&=\sum y_{E,i}\cdot f_{i}\left( S_{1},...,S_{N}\right) \cdot V-r_{E}\cdot E,\nonumber \\ \dot{V}&=y_{V}\cdot \left( r_{E}\cdot E-m\cdot V\right) . \end{aligned}$$

The functions $f_{i}$ are substrate specific uptake rates and the yield coefficients $y_{E,i}$ define the amount of energy extracted into the energy reserve per consumed unit of substrate. Independently of the specific substrate, the reserve mobilization rate $r_{E}$determines the energy channeled from cellular reserves into growth and maintenance expenditures^24^. The coefficient *m* defines the maintenance costs required per unit structural biomass and $y_{V}$ is the biomass yield per unit energy.

As long as substrate is available, the energy reserve *E* is replenished by catabolic processes, extracting energy from the available substrates at rates, which depend on the different substrate concentrations. Here, we assume the general form

2 $$\begin{aligned} f_{i}\left( S_{1},...,S_{N}\right) =\frac{\mu _{i}S_{i}}{K_{i}+S_{i}+\sum _{j\ne i}a_{i,j}S_{j}}, \end{aligned}$$

where $\mu _{i}$ is the maximal uptake rate for the *i*-th substrate, which is approximated for $S_{i}\rightarrow \infty$, and $K_{i}$ is the half-saturation constant of the *i*-th substrate. The matrix $\left\{ a_{i,j}\right\}$ is the interaction matrix, containing inhibition coefficients $a_{i,j}\ge 0$ that describe density-dependent inhibitory relationships between the different substrates.

Together, the coefficients $$a_{i,j}$$ can be understood as a directed regulatory network, as shown in the illustrative example in Fig. 5(b). Each inhibition coefficient $$a_{i,j}$$ determines how strong the uptake of substrate $$S_i$$ is inhibited by the presence of substrate $$S_j$$. We assume that interactions do not form recurrent, inhibitory cycles, e.g., substrates do not have a negative influence on their own uptake ($$a_{i,i}=0)$$, nor do we allow two substrates to be mutually inhibitory (see the next section). Otherwise, we do not impose restrictions on the topology of the interaction network: the uptake of a substrate may be inhibited by one (e.g., $$S_1$$ and $$S_7$$ in Fig. 5(b)) or several other substrates ($$S_4$$ and $$S_6$$), the substrate may not be inhibited at all ($$S_8$$), it may inhibit the uptake of several others ($$S_2$$ and $$S_5$$), and it may at the same time be inhibited by several substrates and expose an inhibitory influence on others ($$S_3$$). See SI Section S1 for a simple example.

### Topology of the substrate inhibition network

When considering the network defined by the inhibition coefficients $a_{i,j}$, mutually inhibiting configurations (that is, substrates inhibiting each others uptake) can probably be neglected, because in a natural situation such a mutual inhibition would be a severe disadvantage for any organism. Such recurrent inhibitions are not restricted to the simple case of direct reciprocal inhibitions between two substrates. They may as well arise from indirect interaction between substrates that are mediated via directed paths in the network. In network terminology, a directed path corresponds to a sequence of interactions between pairs of substrates such that the effected substrate of each interaction in the sequence is the same as the affecting substrate of the next interaction in the sequence. For example, the sequence $S_2 \rightarrow S_3 \rightarrow S_4$ constitutes a directed path in the example network in Fig. 5(b). Due to indirect interactions, all substrates downstream a directed path are potentially affected by inhibition. As a consequence, to avoid inhibitory deadlocks, directed paths in the substrate inhibition network should never lead back to the first substrate in the path. In other words, the inhibition network should not contain closed loops, or, in network parlance, the structure of inhibitory substrate interactions should constitute a directed acyclic graph (DAG)^40^.

**Fig. 6:**
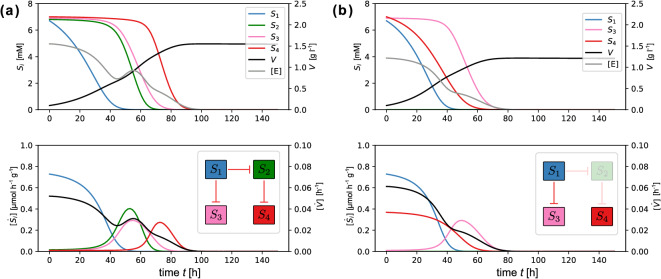
Substrate utilization in an exemplary four-substrate environment with inhibition sequences $S_{1}\rightarrow S_{2}\rightarrow S_{4}$, and $S_{2}\rightarrow S_{4}$ (see network structure insets). Panel (**a**) shows the sequential utilization of substrates following initial concentrations $\varvec{S}_{0}=\left( S_{i}\left( 0\right) \right) _{i=1,...,4}=\left( 6.7,6.8,6.9,7.0\right)$. Panel (**b**) shows the disruption of the sequential utilization in absence of substrate $S_{2}$, i.e, when setting $S_{2}\left( 0\right) =0.0$ while keeping all else as in Panel (a). Uptake parameters: $\varvec{\mu }=\left( 1.0,0.8,0.5,0.5\right)$, $\varvec{y}_{E}=\left( 40.0,25.0,23.0,18.0\right)$, $\varvec{K}=\left( 2.5,2.5,2.5,2.5\right)$; growth parameters are $y_{V}=0.002$, $r_{E}=0.35$, $m=0.05$.

These ideas are illustrated in Figure 6, which shows the dynamics generated by model (1) in a directed acyclic graph with inhibition sequences $S_1 \rightarrow S_2 \rightarrow S_4$ and $S_1 \rightarrow S_3$. The graph does not contain a closed loop as there is no inhibition leading back from $S_4$ to $S_1$, so a deadlock situation is avoided. The parameters for this experiment are chosen such that the highest utility is provided by substrate $S_{1}$, followed by substrates $S_{2}$ and $S_{3}$, which have similar characteristics, and lastly, the low-quality substrate $S_{4}$. If all substrates are present initially at equal concentrations [see Fig. 6(a)], substrate $S_{1}$ is consumed first, followed by a diauxic shift in the simultaneous consumption of $S_{2}$ and $S_{3}$, and finally a second diauxic shift in the consumption of $S_{4}$, as soon as $S_{2}$ is depleted. Thus, the inhibition sequences of substrates in the interaction network are reflected in the model outcome by a corresponding sequential utilization of substrates.

This observations suggests that the topology of the interaction network is related to a priority ranking in the consumption of substrates, and indeed, any directed acyclic graph can be associated with a partial order on the graph’s nodes, here the substrates^40^. For the example shown in Figure 6, the inhibition sequences induce the partial order $\mathcal{O}=\left\{ S_1>S_2>S_4,\,S_1>S_3\right\}$. Such an ordering makes sense from a physiological point of view: If potential substrates differ in their utility for the organism, they are naturally ordered by this intrinsic quality. In such a situation it might be beneficial for the consumer to prioritize the take-up of higher quality substrates, which could be regulated via a directed acyclic inhibition network in which the associated (partial) prioritization order of substrates corresponds to their intrinsic order in utility – which is exactly the situation shown in Fig. 6.

The association between the topology of the inhibition network and the dynamic outcome is not trivial, however, because the level of inhibition depends not only on the inhibition coefficients $a_{ij}$, but also on substrate concentrations. Thus, a sequence of substrate inhibitions can be broken if certain substrates have a small initial concentration to begin with. This is illustrated in Fig. 6 where the sequence of substrate utilization depends on the initial presence of substrates. As shown in Panel (b), if $S_{2}$ is not supplied, $S_{4}$ is lacking an inhibitor and, in contrast to Panel (a), is consequently consumed together with $S_{1}$ from the beginning on; while finally, $S_{3}$ is used after all of $S_{1}$ has been taken up. Graph-theoretically, this dependence on initial substrate concentrations in the inhibition network of Fig. 6 can be understood from the fact that the corresponding prioritization order of substrates is not strict. In general, a prioritization is denoted to be strict if the directed acyclic graph is maximal with respect to the order. That is, if for all substrates $S_{i}$ and $S_{j}$ with $S_{i}<S_{j}$ according to the order, $S_{j}$ inhibits the uptake of $S_{i}$. In the example, shown in Figure 6 this strictness is violated because a maximal directed acyclic graph would additionally contain the inhibition $S_1 \rightarrow S_4$, which is absent in the example network.

It should be noted that these more theoretical considerations may surpass what is realized in natural systems. As each inhibitory mechanism may be assumed to be associated to a dedicated genetic complex and involves the synthesis of its chemical agents by the cell, it usually implies a metabolic cost. For instance, the physiological implementation of a strict prioritization order on several substitutable substrates would come at a considerable expense, which might make it unlikely to occur naturally.

### Parameter fitting and detection of inhibitory regulations

In order to detect substrate interactions in experimental timelines of bacterial population growth, we aim to detect inhibitory interactions $a_{i,j}>0$ between present substrates, *cf.* Eq. (2). For this, we compare the quality of fits of simulated growth curves from model (1) to experimental data for different sets of substrate interactions $a_{i,j}>0$ in a systematic approach.

#### Clustering of similar substrates

If two substrates, $S_{j}$ and $S_{\ell }$, feature similar depletion curves, the optimal values for the interactions $a_{i,j}$ and $a_{i,\ell }$ with another substrate *i* will be weakly determined since one interaction may be replaced by the other giving a similar quality of fit. In such cases it is impossible to discriminate possible inhibitory effects induced by either *j* or $\ell$ if no further information is available. Perfect similarity in this context means that the observed concentration time lines $s_{\ell }\left( t_{k}\right)$ and $s_{j}\left( t_{k}\right)$ of two substrates are connected by a simple scaling

3 $$\begin{aligned} s_{\ell }\left( t_{k}\right) \approx \beta \cdot s_{j}\left( t_{k}\right) \end{aligned}$$

for all measured time points $t_{k}$ with $k=1,..,K$ and some constant $\beta \ne 0$. Then, a full model fit would fail in uniquely identifying the coefficients $a_{i,j}$ and $a_{i,\ell }$ since the residuals resulting from choosing, for instance, different coefficients $a_{i,j}^{\prime }=a_{i,j}+\beta a_{i,\ell }$ and $a_{i,\ell }^{\prime }=0$, would be the same. Thus, in the present framework, there is a fundamental limitation to the discrimination of physically inhibiting substrates that have similar depletion curves according to Eq. (3).

Speaking more generally, the interaction coefficients of substrates satisfying Eq. (3) imply a lack of identifiability^29^, because changes in one parameter can be compensated by changes in another yielding the same model output. This implies a low confidence for the estimation of the individual parameters values, i.e., the inverse problem is ill-conditioned. Such a situation can be handled by introducing equivalence classes of parameters, which cannot be uniquely determined and introduce a single aggregate parameter for these^41^.

We follow this approach and summarize substrates into clusters of similar temporal characteristics before a fitting is executed, such that within each cluster substrates satisfy the relation (3). For two substrates, $\ell$ and *j*, of the same cluster, we thus introduce the constraint $a_{i,\ell }=a_{i,j}$. To perform the clustering, we consider the following measure of dissimilarity on substrate timelines:

4 $$\begin{aligned} d\left( S_{j},S_{i}\right) :=\inf _{\beta \in \mathbb {R}_{>0}}\left\{ \frac{2\cdot \sum _{k=1}^{K}\left( S_{j}\left( t_{k}\right) -\beta S_{i}\left( t_{k}\right) \right) ^{2}}{\beta ^{2}\sum _{k=1}^{K}\left( S_{i}\left( t_{k}\right) -\bar{S}_{i}\right) ^{2}+\sum _{k=1}^{K}\left( S_{j}\left( t_{k}\right) -\bar{S}_{j}\right) ^{2}}\right\} . \end{aligned}$$

Assuming that the substrate concentrations are not constant, and there exists a time $t_k$ where both are present, i.e., $S_{i}\left( t_{k}\right) \cdot S_{j}\left( t_{k}\right)>0$, the infimum is a minimum and the distance fulfills the following criteria (*cf.* SI Sec. S4): (i) it is symmetric, i.e., $d\left( S_{j},S_{\ell }\right) =d\left( S_{\ell },S_{j}\right)$, (ii) two substrates satisfying Eq. (3) have maximal similarity, i.e., $d\left( S_{j},S_{\ell }\right) \approx 0$, and (iii) the similarity is invariant under scaling of either substrate, i.e., $d\left( S_{\ell },S_{j}\right) =d\left( c\cdot S_{\ell },S_{j}\right)$, meaning that the absolute concentration of a substrate’s timeline does not matter. Likewise, for two substrates satisfying Eq. (3), the optimal interaction coefficients $a_{i,j}$ and $a_{i,\ell }$ to all other substrates can be chosen identically and one may treat all substrates of a similarity cluster by a single state variable.

For the *P. inhibens* experiments, a hierarchical clustering [Fig. 1(b)] was obtained by employing a farthest point cluster distance, evaluated across all four experimental replicates. The set of clusters was obtained by a cut height of 0.075 as indicated by the dashed line in Fig. 1(b). The choice of an appropriate cut height depends on the specific problem. Here, we chose a maximal height, which resolves the time series of mannitol and N-acetylglucosamine into different clusters in order to distinguish their specific influence on glucose depletion.

#### Componentwise fitting of substrate related parameters

Even if we do not take the interaction coefficients $a_{i,j}$ into account, the full model (1) has $3N+3$ parameters: Namely, for each of the *N* substrates the three substrate-specific parameters $y_{E,i}$, $\mu _{i}$, $K_{i}$, and the overall growth parameters $r_{E}$, $y_{V}$, and *m*. Thus, as the number of substrates increases, the number of parameter values also grows substantially, which can make parameter fitting challenging. Attempting to fit all parameter values simultaneously using numerical optimization routines may be infeasible for a large number of substrates.

In order to overcome this limitation we use a componentwise fitting procedure, which fits the uptake function [Eq. (2)] for each substrate, resp. substrate cluster, individually. This is done by replacing dependent variables $S_{j}(t)$, $j\ne i,$ and *V*(*t*) on the right-hand side of Eq. (1) by an interpolation of the corresponding observed values for the substrate concentrations, $s_{j}(t)$, and the biomass, *v*(*t*). This decouples the dynamics among substrates and reduces the problem to fitting *N* independent simplified systems

5 $$\begin{aligned} \dot{S}_{i} \left( t\right) =-f_{i}\left( s_{1}\left( t\right) ,...,s_{i-1}\left( t\right) ,S_{i}\left( t\right) ,s_{i+1}\left( t\right) ,...,s_{N}\left( t\right) \right) \cdot v\left( t\right) . \end{aligned}$$

Based on this equation, the parameter values of the uptake functions $f_i$, *cf.* Eq. (2), i.e., the maximal uptake rate $\mu _{i}$, the half saturation constant $K_{i}$, and eventually the inhibition coefficients $a_{i,j}$ affecting the uptake of substrate *i*, can be estimated.

We identify an appropriate set of non-zero interactions $a_{i,j}$ to be included into the model by determining the most significant interactions incrementally. That is, we first assume for each substrate an influence of a chosen single substrate or substrate cluster and determine whether the inclusion of the corresponding interaction parameter improves the fit to the experimental data significantly. In the next step, two interactions are considered. Theoretically, this procedure may be continued to an arbitrary number of interactions, though in the exemplary application to *P. inhibens* growth experiments including more than one substrate cluster did not result in any further improvements (see section “Results”).

In order to assess the quality of the fit we consider a square error function *F* that penalizes deviations between the model and observations at the measurement times $t_{k}$, $k=1,...,k_{\textrm{end}}$. This error function serves two purposes. Firstly, we aim to approximate observed substrate concentrations $s_{i}$ by the modeled values $S_{i}$. Secondly, as uptake rates can sometimes reveal sequential patterns more clearly (see, e.g., Fig. 6), we attempt to minimize deviations between modeled $[\dot{S}_{i}]$ and observed $\left[ \Delta s_{i}\right]$ specific uptake rates, which are determined as

$$\begin{aligned} [\dot{S}_{i}]_{k}=\frac{2\dot{S}_{i}\left( \frac{t_{k}+t_{k+1}}{2}\right) }{\left[ v_{i}\left( t_{k}\right) +v_{i}\left( t_{k+1}\right) \right] } \text { and } \left[ \Delta s_{i}\right] _{k}=\frac{2\left[ s_{i}\left( t_{k+1}\right) -s_{i}\left( t_{k}\right) \right] }{\left[ v_{i}\left( t_{k}\right) +v_{i}\left( t_{k+1}\right) \right] \Delta t_{k}}. \end{aligned}$$

This suggests an objective function *F* of the form

6 $$\begin{aligned} F=w_{1}F_{1}\left( s_{i};S_{i}\right) +w_{2}F_{2}\left( \left[ \Delta s_{i}\right] ;[\dot{S}_{i}]\right) , \end{aligned}$$

where $w_{1}=1/\max _{k} s_{i}\left( t_{k}\right)$ and $w_{2}=1/\max _{k\ge k_{0}} \left[ \Delta s_{i}\right] _{k}$ are normalizing weights for the balanced penalization of the total concentration error

$$\begin{aligned} F_{1}\left( s_{i};S_{i}\right) =\sum _{k=1}^{k_{\textrm{end}}}\left( S_{i}\left( t_{k}\right) -s_{i}\left( t_{k}\right) \right) ^{2}, \end{aligned}$$

and the specific rate error

$$\begin{aligned} F_{2}\left( \left[ \Delta s_{i}\right] ;[\dot{S}_{i}]\right) =\sum _{k=k_{0}}^{k_{\textrm{end}}}\left( [\dot{S}_{i}]_{k}-\left[ \Delta s_{i}\right] _{k}\right) ^{2}. \end{aligned}$$

Here, $k_{0}$ defines a lower bound to the time interval where it is feasible to construct the specific rates, which can be very volatile for the initially low biomass concentrations in incubation experiments.

In practice, experiments are often repeated or run in parallel several times to ensure reproducibility of results. To maximize the information gained from such experiments, it is recommended to fit all experimental runs simultaneously using a shared set of uptake parameters and a composite objective function, which is the sum of multiple error functions of the form (6) – one for each replicate.

### Experimental data

We tested the proposed framework on data from a laboratory experiment with the marine heterotrophic bacterium *Phaeobacter inhibens* DSM 17395, which is an aerobic heterotrophic representative of the *Roseobacter *group and is known to possess versatile catabolic and complex regulatory capacities^23,42^.

In the experiment, the growth of *P. inhibens* on a mixture of organic substrates was measured, as described by Wünsch et al.^21^ For each tested substrate condition, four bioreactors were operated as a batch culture in parallel in a well-mixed glass vessel under controlled conditions of culture volume, temperature, pH, dissolved oxygen tension, and growth medium. After inoculated culture cells had adapted their growth to the medium by three passages under identical conditions, the defined starting medium was supplemented with a mixture of organic substrates. The contained substrates were: D-glucose (Glc), D-mannitol (Man), N-acetyl-D-glucosamine (Nag), L-histidine (His), L-leucine (Leu), L-lysine (Lys), L-phenylalanine (Phe), L-tryptophan (Trp), L-threonine (Thr), L-valine (Val), and isoleucine (Ile), see SI Fig. S4.

For each of the four replicate measurements, high-resolution growth curves were provided from measurements of substrate depletion, optical density (OD) and cellular dry weight (CDW) during growth as a function of incubation time (see Fig. 1). Theoretical ATP yield was computed based on recently reconstructed catabolic pathways for each substrate. The resulting values are shown in SI Table S1. Wünsch et al.^21^ give a detailed description of the experiment.

## Supplementary Information

Supplementary Information.

## Data Availability

All data and code necessary to reproduce the results presented in this article are available at https://github.com/leoluecken/growth-on-mixtures-analysis. The repository includes the full set of scripts and documentation to support reproducibility.
