# Supplementary material for: A network-based analysis of bacterial growth on substrate mixtures uncovers glucose inhibition in Phaeobacter inhibens
Source: Sci Rep. 2026 Jan 8;16:1289. doi: 10.1038/s41598-025-33583-6 (PMC12791127; doi:10.1038/s41598-025-33583-6)
Supplement: Supplementary file 1 — Supplementary Information. [file 41598_2025_33583_MOESM1_ESM.pdf]

Supporting Information

A Network-Based Analysis of Bacterial Growth on Substrate Mixtures Uncovers Glucose Inhibition in *Phaeobacter Inhibens*

Leonhard Lücken[1] and Bernd Blasius[1,2]

[1] Institute for Chemistry and Biology of the Marine Environment (ICBM),
Carl von Ossietzky Universität Oldenburg, Germany.
[2] Helmholtz Institute for Functional Marine Biodiversity, 26129 Oldenburg, Germany.

S1 Inhibitory configurations for growth on two substrates

This section illustrates the effect of inhibitory interactions in the most simple setup of only two substrates. Figure S1 shows the corresponding time series, obtained from simulating model (1) of the main text for a batch culture with two substrates, S_A and S_B, for different configurations of the interaction matrix.

Panel (a) shows a scenario without any uptake inhibitions, i.e., the case of independent substrate uptake of both substrates, $a_{i,j} \equiv 0$. Following the standard dynamics of a batch reactor [1, 3], both substrates are declining from their initial concentrations as they are taken up by the consumer, building up structural biomass until the system reaches a stationary state when both substrates are depleted. Initially, both substrates are taken up at a rate close to the respective maximal rate μ_i, $i = A, B$, as long as their concentrations are significantly higher than the corresponding half saturation values K_i. The depletion of substrate S_A proceeds slightly slower than that of substrate S_B, because the latter is modeled to possess a higher utility ($\mu_A < \mu_B$, $K_A > K_B$, and $y_{E,A} < y_{E,B}$, see caption of Fig. S1 for the exact values of all parameters).

Panel (b) shows the system's dynamics observed for an inhibition of the uptake of the low-quality substrate S_A by the higher-quality substrate S_B with strength $a_{A,B} = 50$. As the inhibitory effect is strong, the uptake of substrate S_A is effectively suspended as long as non-vanishing amounts of S_B are present and only sets in after depletion of S_B to very small concentrations. The resulting diauxic growth is most clearly seen when one considers the specific

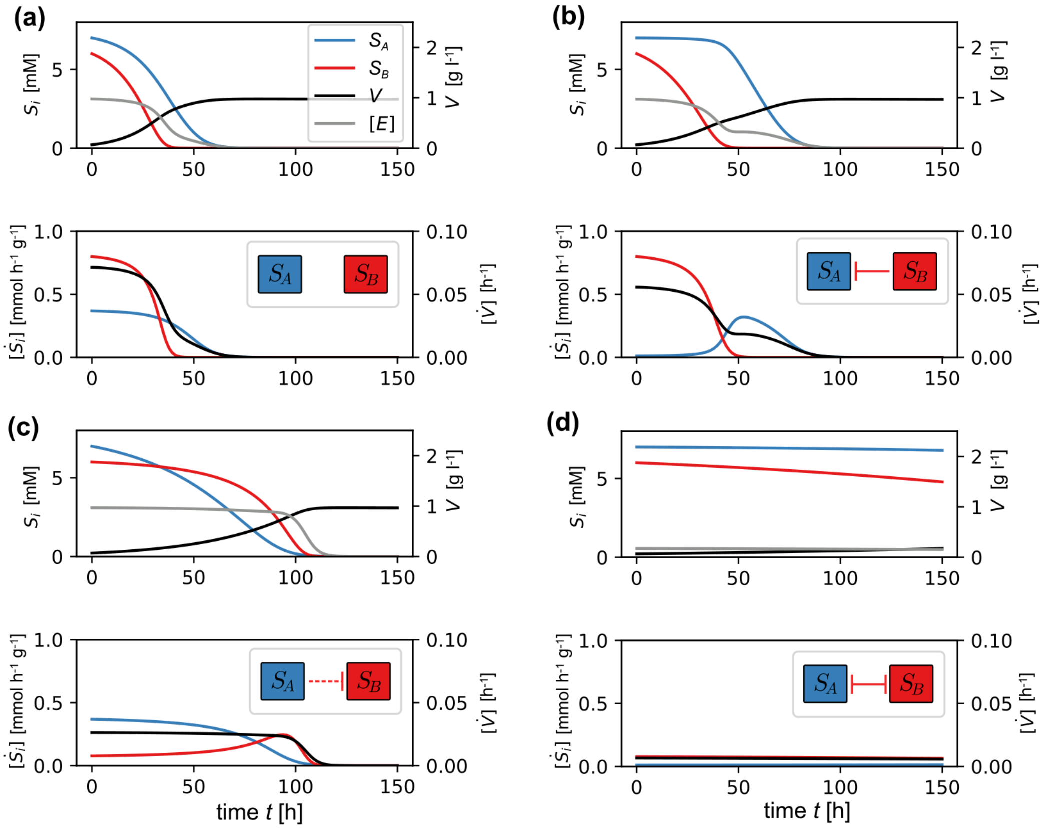

Figure S1: Effect of different inhibitory interactions in the growth model (1) of the main text with two substrates S_A and S_B. The solid lines in the upper panels show the time courses of the substrate concentrations, the concentration of structural biomass V (black) and the specific concentration of the energy reserve $[E] = E/V$ (gray). The lower panels show specific substrate uptake rates, $[\dot{S_A}] = \dot{S_A}/V$(blue) and $[\dot{S_B}] = \dot{S_B}/V$ (red), and the specific growth rate $[\dot{V}] = \dot{V}/V$ (black). (**a**) Independent consumption of substrates $a_{i,j} \equiv 0$; (**b**) inhibition of substrate S_A by substrate S_B with $a_{A,B} = 50$; (**c**) inhibition of substrate S_B by substrate S_A with $a_{B,A} = 10$; (**d**) mutual inhibition with $a_{A,B} = a_{B,A} = 50$, leading to a closed loop $S_A \to S_B \to S_A$ in the inhibition network. Uptake parameters are $\mu_A = 0.5$, $\mu_B = 1.0$, $K_A = 2.5$, $K_B = 1.5$, $y_{E,A} = 30$, $y_{E,B} = 40$; growth parameters are $y_V = 0.002$, $r_E = 0.35$, $m = 0.05$; initial substrate concentrations are $S_A(0) = 7$, $S_B(0) = 6$, initial biomass is $V(0) = 0.1$ and initial reserve density $E(0)$ is chosen such that $\dot{E} = 0$, when using the initial substrate and biomass values in model (1) of the main text.

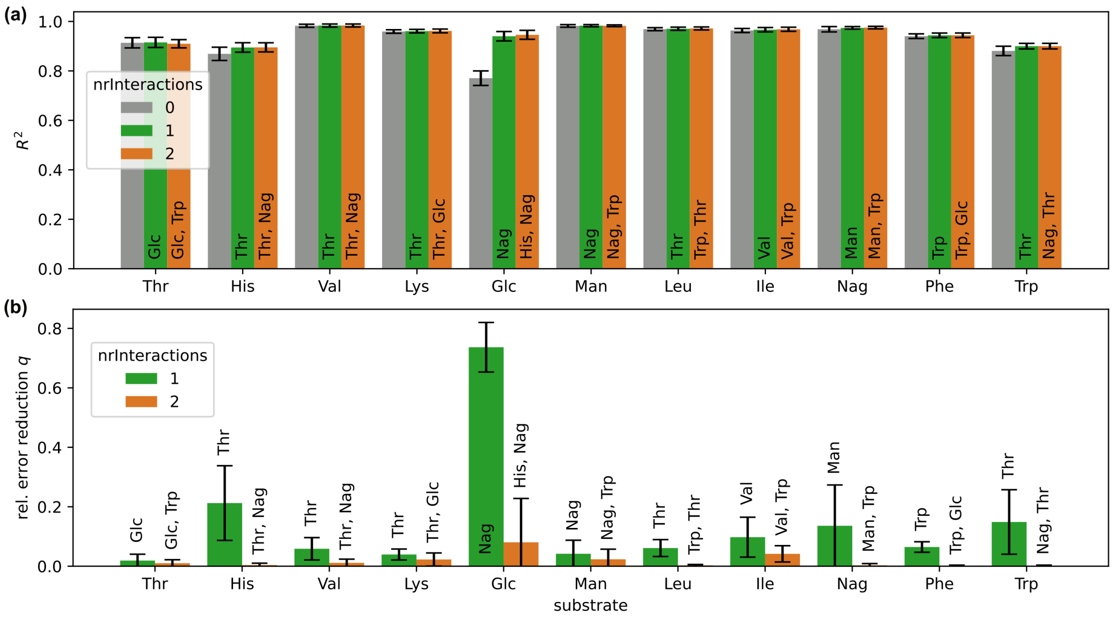

Figure S2: Overview of model fits for different substrates and and number of substrate interactions. (a) Explained variance R^2 [Eq. (S1)] for fitted uptake parameters of different substrates. Grey bars: no interactions; green bars: optimal fit for inhibition by one substrate or substrate cluster; orange bars: optimal fit for inhibition by two substrates or substrate clusters. (b) Relative reduction of errors q corresponding to (a) [Eq. (S2)].

growth and uptake rates in the lower plot of Panel (b), showing the sequential uptake of the two substrates and the corresponding different growth rates. Panel (c) shows the time course for the opposite case that the high-quality substrate S_B is inhibited by low-quality substrate S_A, but with a lower inhibition strength $a_{B,A} = 10$ than in case (b). It can be observed, that S_B is utilized at a low, but non-vanishing rate even at initially high concentration of S_A. Still, since the total growth rate is significantly smaller than in case (a), the substrate depletion of S_A is slow and delayed. Interestingly, even though the uptake of S_A is not inhibited here, its depletion is still delayed compared to case (a) because the slower overall build-up of structural biomass. Finally, panel (d) illustrates the deadlock situation arising from a mutual inhibition of both substrates (with symmetric inhibition coefficients $a_{A,B} = a_{B,A} = 50$). Here, both substrates are taken up at very low rates for a long time since the presence of one substrate impedes the utilization of the other.

S2 Supplemental material on fitting *P. Inhibens* experiments

S2.1 Error reduction

For each substrate fraction of explained variance was calculated as

$$R^2 = 1 - \frac{F}{\bar{F}} \in [0,1]\,, \tag{S1}$$

with the square error F defined in Eq. (6) of the main text. The denominator $\bar{F}$ was computed as the value of the square error obtained by inserting constant mean value approximations for S and $\dot{S}$.

To quantify the improvement induced by including an additional interaction with respect to a base model, we computed the relative error reduction

$$q = \frac{F - F'}{F}, \tag{S2}$$

where F, and F', are the errors of a base model, and the extended model, respectively.

The maximal R^2-values per substrate and per number of non-zero inhibition coefficients are reported in Fig. S2(a). In the plot, different bar colors correspond to a different number of inhibitory actors upon the corresponding substrate. For each substrate and number of interactions, the maximal value R^2 among all corresponding inhibitory combinations determines the height of the bar and the maximizing inhibitor is indicated on the bar.

Figure S2(b) shows the maximal error reduction for the different substrates among those fitted with an additional interaction. Here, green bars indicate the maximal relative error reduction q due to including a single interaction with respect to a model without interactions, and orange bars indicate the maximal relative error reduction for including two interactions with respect to the optimal model with one interaction.

S2.2 Fitting the growth-related subsystem

Similar to the fitting of the specific uptake parameters for each substrate [*cf.* Eq. (5) of the main text], we fit the growth parameters m, r_E , and y_V for the subsystem

$$\dot{E} = \sum_i y_{E,i} \cdot f_i\left(\hat{s}_1, ..., \hat{s}_N\right) \cdot V - r_E \cdot E, \tag{S3a}$$

$$\dot{V} = y_V \cdot \left(r_E \cdot E - m \cdot V\right). \tag{S3b}$$

The energy yields $y_{E,i}$ are fixed to theoretically derived values listed in Table S1. We refer to Wünsch et al. [4] for details. Further, the uptake parameters obtained from fitting the different uptake subsystems [Eq. (5) of the main text] to define the different uptake response functions $f_i\left(\hat{s}_1, ..., \hat{s}_N\right)$. Here, the interpolations $s_i(t)$ of the observed substrate concentrations

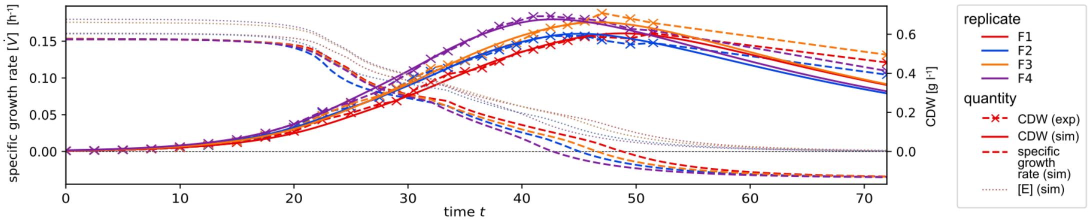

Figure S3: Comparison of experimental growth curves and trajectories generated by (S3) with parameters $m = 12.34$, $r_E = 0.5$, $y_V = 1.4 \cdot 10^{-3}$, and $V_0 = (1.25, 1.99, 1.72, 2.27) \cdot 10^{-3}$. No substrate inhibitions were considered. Different colors refer to the different experimental replicates, dotted lines with crosses correspond to observed cell dry weight, solid lines to simulated biomass V, dashed lines to simulated specific growth rate $[\dot{V}] = \dot{V}/V$, and dotted lines to simulated specific reserve density $[E] = E/V$.

are treated as external model input.

We define a square error function for the biomass component analogously to Eq. (6) of the main text as

$$F = w_1 F_1 (v; V) + w_2 F_2 \left([\Delta v] ; [\dot{V}] \right), \tag{S4}$$

with $w_1 = 1/\max_k v_i (t_k)$ and $w_2 = 1/\max_{k \geq k_0} [\Delta v_i]_k$.

Trajectories for all experimental replicates generated by the optimal fit for the growth model [Eq. (S3)] are shown in Figure S3. Here, the only replicate specific parameter is the initial value $V(0) = V_{0,r}$, $r = 1, ..., 4$, for the biomass, while the parameters m, r_E, and y_V, were fitted for all replicates simultaneously. Treating $V_{0,r}$ as parameters is important to account for the sensitive dependence of the growth trajectory on small variations of this value. The optimization of the square error functional (S3) yields a fraction of explained variance $R^2 = 0.96$ for the decoupled growth model (S3) with parameter values given in the caption to Fig. S3.

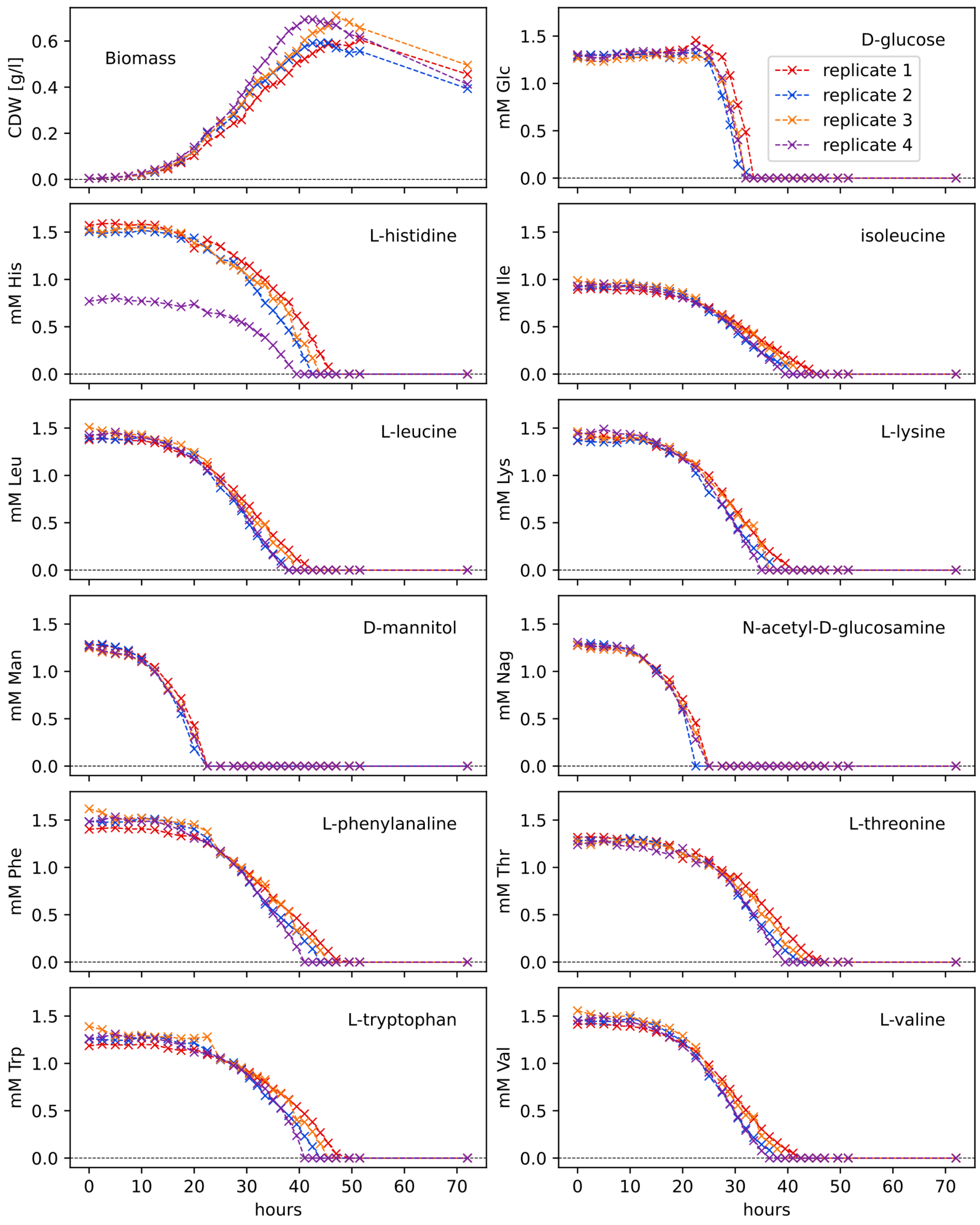

Figure S4: Experimental data for the growth of *P. Inhibens* in batch reactors containing a mix of carbon sources. Each panel shows data for one measured value from four replicate experiments. The upper left panel shows the cell dry weight while the other 11 panels show concentration timelines for the different substrates. Data by Wünsch et al. [4]

.

Table S1: Theoretically derived substrate specific values for the ATP yields $y_{E,i}$ [mM ATP/mM] of different substrates, see [4].

	Thr	His	Val	Lys	Glc	Man	Ile	Leu	Nag	Phe	Trp
$y_{E,i}$	22.2	26.6	31.9	34.4	36.6	39.6	40.7	40.7	47.4	48.7	60.6

S3 Comparison of cultures growing on mixed and individual carbon sources

Growth curves of *Phaeobacter Inhibens* and substrate concentration curves for batch cultures on individual carbon sources are shown in Figure S5 for all substrates except isoleucine, on which *P. Inhibens* did not grow [4].

In Figure S6 we show the proportional changes when comparing the growth on an individual substrate with growth on the mixture for: (i) the maximal uptake rate μ_0 (resp. μ_{mix}) and (ii) the effective uptake rate $r_{1,0} = \mu_0/(K + 1\,\text{mM})$ (resp. $r_{1,\text{mix}} = \mu_{\text{mix}}/(K + 1\,\text{mM})$) at a substrate concentration of 1 mM. That is,

$$\Delta\mu = \frac{\mu_{\text{mix}} - \mu_0}{\mu_0}, \text{ and } \Delta r_1 = \frac{r_{1,\text{mix}} - r_{1,0}}{r_{1,0}}.$$

In Panels (a)-(c) of Fig. S6, we observe a significant reduction in individual maximal uptake rates for all substrates when comparing fitted values from growth on individual substrates versus the mixture, with the most pronounced effects seen for threonine and glucose. To identify systematic relationships between substrate quality and altered uptake characteristics for growth on the mixture, we considered the following indicators derived from cultures on individual substrates:

1. The directly fitted, effective growth rate

$$\gamma = \ln[V(t_1) - V(t_0)]/(t_1 - t_0), \tag{S5}$$

   where $[t_0, t_1]$ is the time interval of the exponential growth phase (SI Fig. S7).

2. Bacterial growth efficiency (BGE) η during the exponential growth phase:

$$\eta = [V(t_1) - V(t_0)]/[S(t_1) - S(t_0)]. \tag{S6}$$

3. ATP yield y_E as computed theoretically by Wünsch et al. (Table S1).

For sugars, we present the relationship between these indicators and glucose uptake inhibition in the main text (see Fig. 2). For amino acids, the change of the uptake rates for the growth on the mixture is not clearly connected to any of these quality indicators. For instance, the maximal uptake of phenylalanine is decreased more strongly than that of, e.g., valine or lysine, although

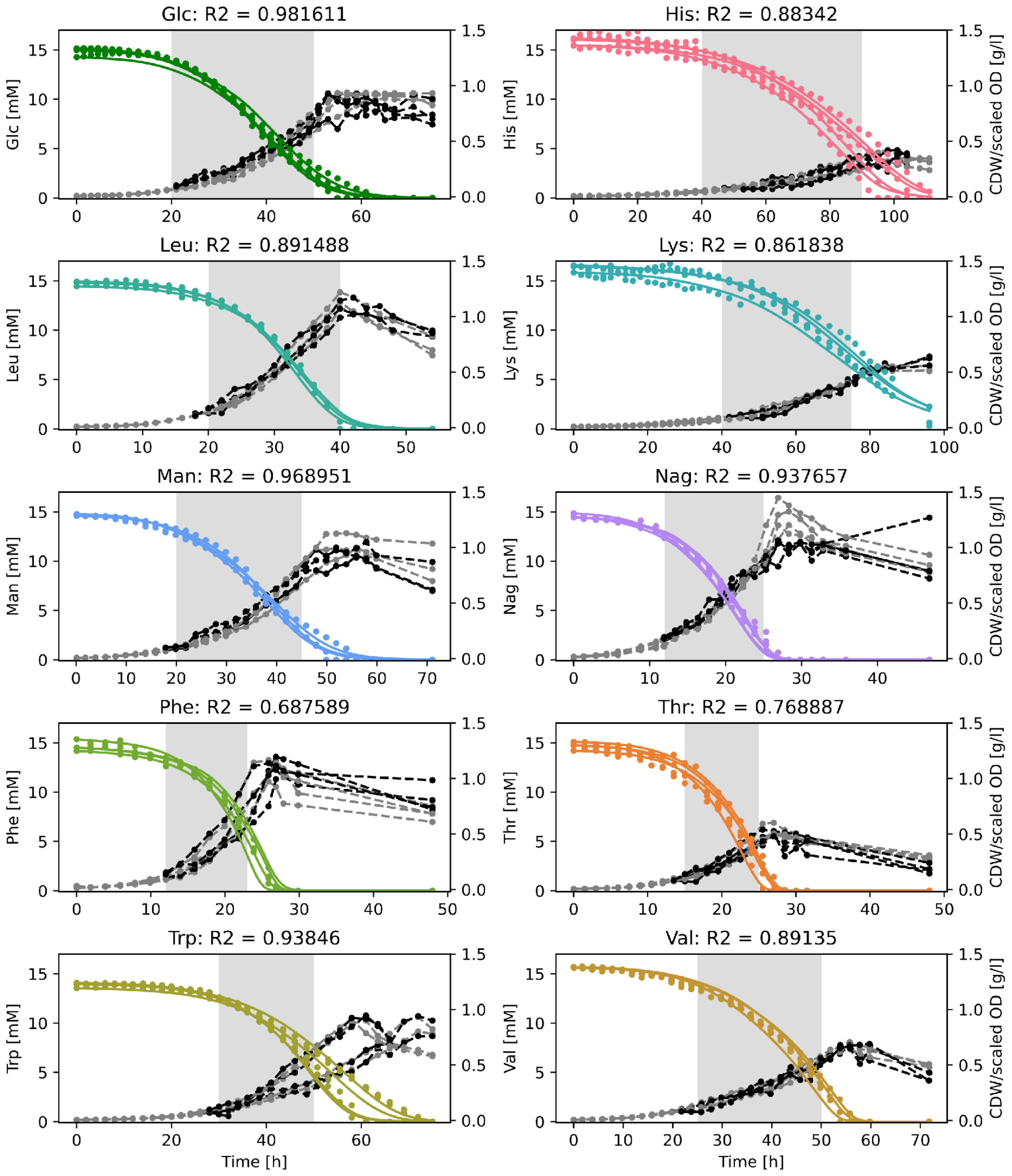

Figure S5: Growth of *P. Inhibens* in batch reactors containing a single carbon source only, as obtained by Wünsch et al. [4]. For each setup three or four replicates were prepared. The colored dots show the measured concentrations, the black and gray dots (connected by a dashed line) show the measured CDW and OD values. Colored continuous curves show the concentrations obtained from the fitted model, the shaded regions are manually defined exponential growth intervals.

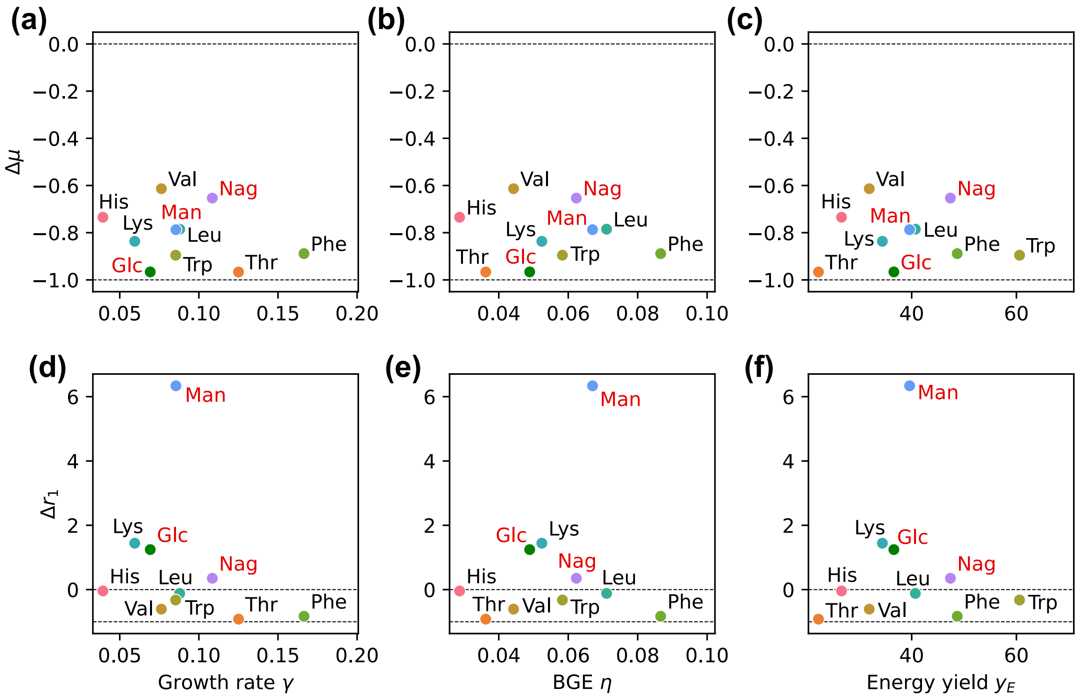

Figure S6: Proportional changes in fitted uptake parameters when growing in a mixture in comparison with characteristics measured for growth on a single carbon source. Sugars are indicated by red labels, amino acids by black labels.

phenylalanine shows higher values for all indicators. In contrast to the changes $\Delta\mu$ of the maximal uptake rates, the changes Δr_1 of uptake rates at substrate concentrations of 1.0 mM are not uniform [*cf.* Fig. S6 (d)–(f)]. For some substrates (most pronounced for mannitol, but also for lysin), the effective rates are increased in the mixture. Because all maximal uptake rates μ decrease, this corresponds to smaller values for the half-saturation constants K, i.e., to a higher substrate affinity μ/K. Excluding glucose as an outlier, the fitted substrate affinity increases approximately tenfold on average. This seems to resemble an adaptation to low/high substrate concentrations along a trade-off between affinity and maximal uptake rates [2]).

S4 Evaluation of interaction detection on surrogate data

To assess the practicality of the proposed protocol for uncovering substrate interactions in mixtures, we generated artificial experimental data employing models with randomized, acyclic inhibition topologies and different types of stochastic perturbations. We then tested the ability of our protocol to uncover the implemented inhibitory interactions between the different substrates.

For the generation of such an artificial experimental system, we prescribed the total number of substrates, the total number of interactions, the intensity of the dynamic noise acting on the

maximal uptake rates μ_i, and the standard deviation of the measurement noise.

The substrate specific uptake parameters $y_{E,i}$, μ_i, and K_i were randomly and independently drawn for each substrate [$K \sim \mathcal{U}(0.001, 2.0)$, $\mu \sim \mathcal{N}_+(0.5, 0.5)$, $y_E \sim \mathcal{N}_+(40.0, 5.0)$]. A prescribed number of randomly chosen interactions were assigned randomly with strengths $a_{i,j} \sim \mathcal{U}(0.0, 50)$. Here, $\mathcal{U}(a, b)$ denotes a uniform distribution on the interval $[a, b]$, and $\mathcal{N}_+(\mu, \sigma)$ denotes a truncated Gaussian distribution, i.e., a normal distribution with standard deviation σ and mean μ, restricted to positive values.

The dynamically perturbed uptake rates μ were modeled as time dependent functions

$$\tilde{\mu}_i(t) = \mu_i + X(t),$$

where $X(t)$ is the trajectory of an Ornstein-Uhlenbeck process with relaxation rate ϑ and volatility σ. That is, a process obeying stochastic dynamics

$$dX(t) = \sigma \cdot dW(t) - \vartheta \cdot X(t) \cdot dt,$$

and $W(t)$ is the generating Wiener process. We use a parameter i_{dyn} to scale the amplitude of the parameter fluctuations away from the mean value μ_i by setting $\vartheta = 0.1/i_{\text{dyn}}$ and $\sigma = 0.1$ for the simulated experimental data. Examples for such surrogate data generated with different values of ϑ are shown in Figure S7.

We studied the separate effects of system size, number of present interactions, dynamic parameter perturbations and measurement noise on the accuracy of the predicted interactions. To this aim, we generated synthetic experimental data sets for different parameter combinations varying one of the above. For each parameter point, we sampled 14 systems and per system, we generated 4 runs, which were used as a basis for parameter fitting. Based on the fit results for different assumed interactions, we predicted the present ones. An interaction was deemed a candidate for a present inhibition if its incorporation implied a relative improvement

$$p = (R^2_{\text{ext}} - R^2)/R^2$$

larger than a threshold θ, where R^2 and R^2_{ext} are the fractions of explained variance [see Eq. S1] associated to the basic fit and the fit including an additional interaction. For each substrate, only the best fit among all with an additional interaction predicted (if it displays $p > \theta$).

To assess the performance of this prediction, we report the overall attained F1-score, recall and precision at each parameter point for a selection of different values of θ [Fig. S8]. The F1-score is a measure of prediction accuracy commonly employed to evaluate detection algorithms by comparing the number of correctly predicted [true positives (TP)], falsely predicted [false positives (FP)] and missed entities [false negatives (FN)]. It balances the importance of

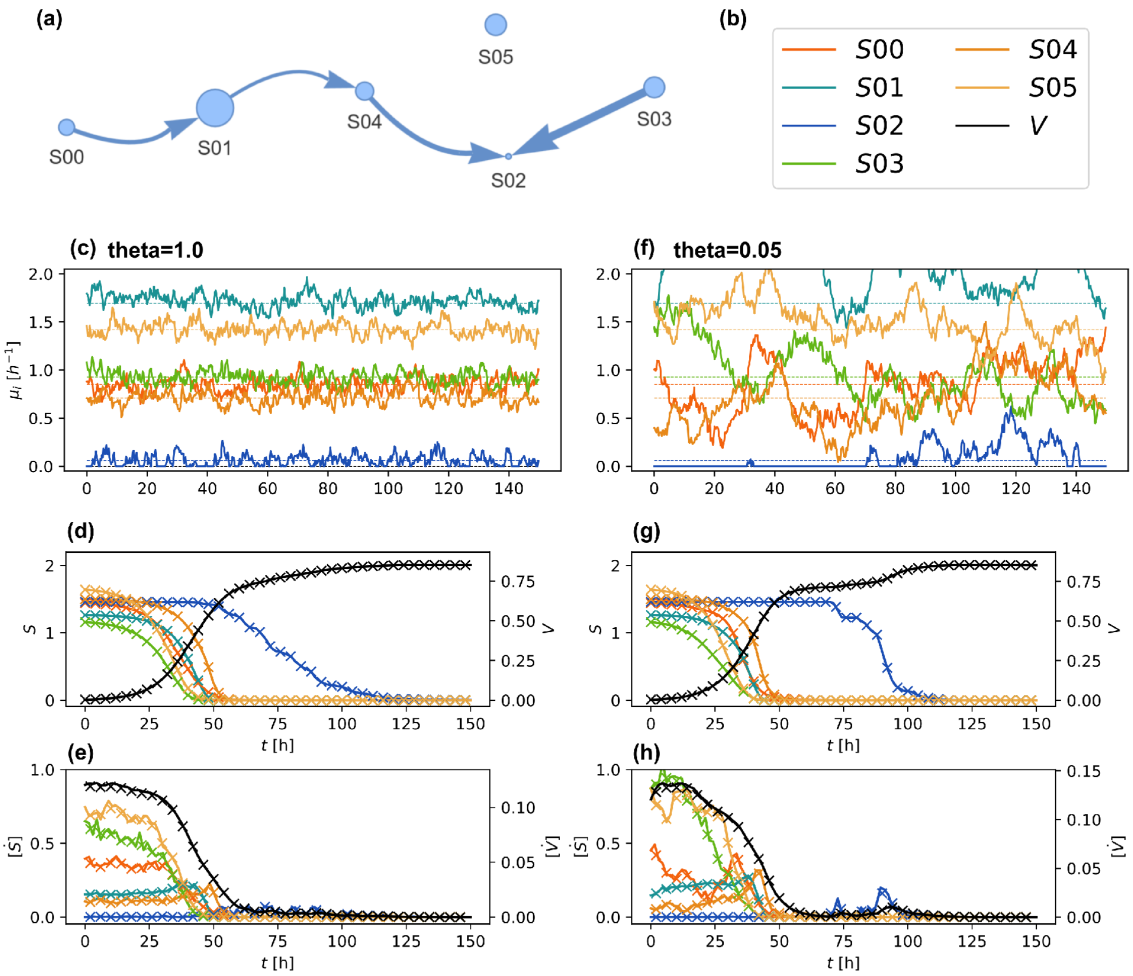

Figure S7: Example for a surrogate experimental trajectory with dynamic noise acting on uptake rates. (a): Randomly generated topology of the inhibitory substrate interaction network [bolder arrows indicate stronger inhibition, larger dots higher uptake rate r_1 , *cf.* Sec. S3]; (b): Color legend; (c), (f): Dynamically perturbed maximal specific uptake rates for higher [(c): $\vartheta = 1.0$] and lower [(f): $\vartheta = 0.05$] noise damping. (d), (g): Substrate concentration and biomass trajectories; (e), (h): Realized total uptake and growth rates.

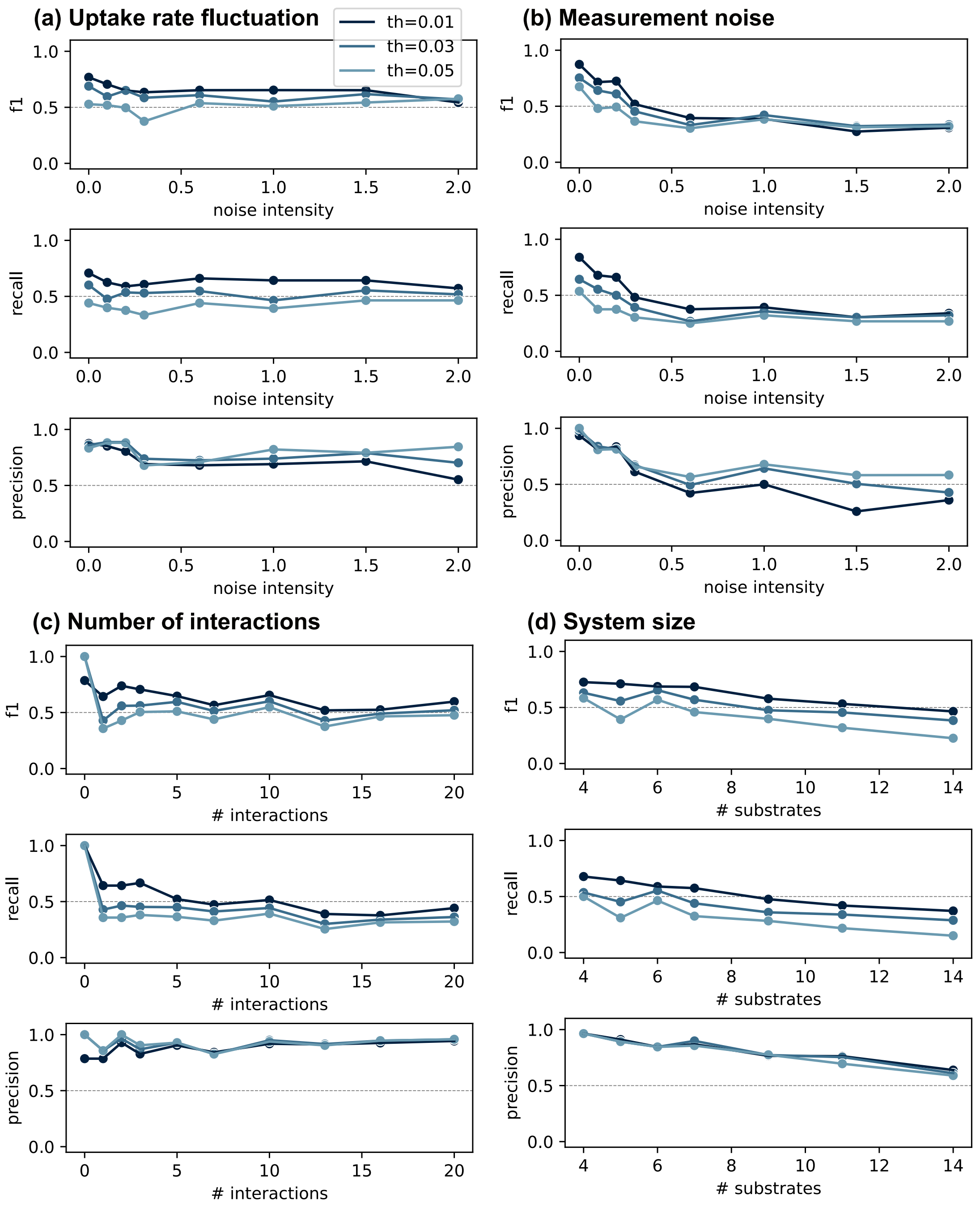

Figure S8: Performance measures for interaction detection using synthetic experimental data (improvement of fit criterion for interaction prediction). The number substrates is 6 and the number of interactions is 4 where not stated otherwise. (a) Different levels of dynamic parameter perturbations; (b) Different levels of measurement noise; (c) Different numbers of present interactions; (d) Different mixture complexities (#interactions = #substrates - 2).

recall = TP/(FN + TP) and precision = TP/(TP + FP) and is defined as

$$\mathrm{F1} = \frac{2 \times \mathrm{precision} \times \mathrm{recall}}{\mathrm{precision} + \mathrm{recall}}$$

As a general pattern, we observe that the accuracy of predictions decreases with higher noise intensity [Fig. S8(a) and (b)] and larger system size [Fig. S8(c) and (d)]. Further, for a fixed parameter value a larger threshold usually increases the precision and decreases the recall as would be expected for a sound detection protocol. In presence of noise [(a) and (b)], these effects seem to balance for the tested thresholds, such that the resulting F1-score is only slightly effected by different choices for θ. In absence of noise [(c) and (d)], the precision is affected more weakly than the recall resulting in highest F1-scores for low thresholds.

We have also tested predicting interactions on the basis of error reduction instead of fit improvement. This alternative criterion is defined as follows. Assume that a basic fit with associated R^2 and a residual mean square error RMSE [i.e., F, see Eq. (6) of the main text] is given and the corresponding values for the system extended by an additional interaction are R^2_{ext} and $\mathrm{RMSE}_{\mathrm{ext}}$. If the relative error reduction

$$q = [(1 - R^2) - (1 - R^2_{\mathrm{ext}})]/(1 - R^2) = (\mathrm{RMSE} - \mathrm{RMSE}_{\mathrm{ext}})/\mathrm{RMSE}$$

achieved by including the additional interaction is larger than θ, the interaction is assumed to be present if it represents the best fit among all with one additional interaction.

The results for this approach are reported in Fig. S9. As for the approach based on fit improvement, precision increases and recall decreases as the threshold increases. However, for this approach, precision and recall are affected to a similar degree, which implies a less uniform impact of θ on the F1-score. For instance, in case (a), the optimal choice is an intermediate threshold. Especially for the cases, where stochasticity [either parameter fluctuations in (a) or measurement noise in (b)] is included, for low thresholds, the low precision dominates the F1-score. In these cases the error of the fitted model cannot vanish completely and, thus, its potential reduction is bounded. Most drastically, this can be seen in (b), where the recall for $\theta = 0.8$ drops to zero already at a measurement noise intensity $i_{\mathrm{obs}} = 0.3$. Hence, to robustly work in the presence of stochasticity (which may be associated to mismatch between the model structure and the modeled process) the detection threshold for error reduction shouldn't be too large.

It is also noteworthy that in the absence of interactions (or for very few interactions), the attained precision was frequently low [*cf.* Panel (c)] for the approach based on error reduction. This means that the error was "significantly" reduced by including a non-existent interaction. In this situation, false positives seem to occur because of a minute improvement of an already very good fit by the inclusion of the interaction. Since the original error is already very small, even a small improvement implies a large relative improvement q of the error, *cf.* (S4). This

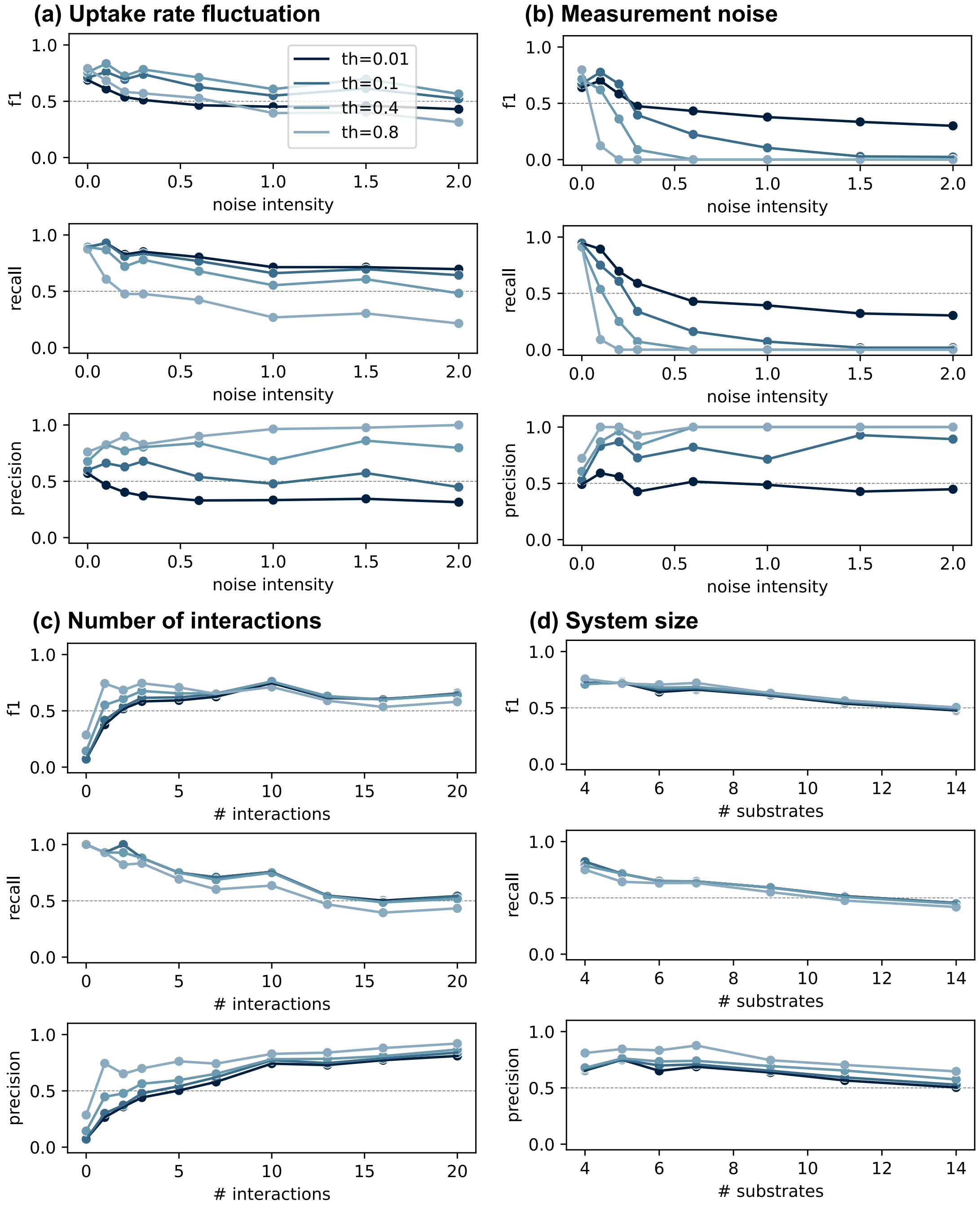

Figure S9: Performance measures for interaction detection using synthetic experimental data (reduction of error criterion for interaction prediction). The number substrates is 6 and the number of interactions is 4 where not stated otherwise. (a) Different levels of dynamic parameter perturbations; (b) Different levels of measurement noise; (c) Different numbers of present interactions; (d) Different mixture complexities (#interactions = #substrates - 2).

problem is less severe in presence of noise since the error will never be very small [*cf.* (a) and (b)]. For many interactions and larger system size [Panels (c) and (d) in Figures S8 and S9], the performance of the error reduction approach is slightly better and more stable than for the fit improvement approach, though. Similarly, for larger systems [case (d)], error reduction seems to be a slightly more reliable indicator than fit improvement.

S5 Dissimilarity of depletion curves

In the main text, we introduced the function

$$d(S_j, S_i) := \inf_{\beta \in \mathbb{R}_{>0}} \left\{ \frac{2 \cdot \sum_{k=1}^K (S_j(t_k) - \beta S_i(t_k))^2}{\sum_{k=1}^K \left(S_j(t_k) - \bar{S}_j\right)^2 + \beta^2 \sum_{k=1}^K \left(S_i(t_k) - \bar{S}_i\right)^2} \right\}$$

to quantify the dissimilarity of substrate depletion curves. This function was defined for the purposes of clustering curves which are merely rescaled versions of each other, i.e., $S_j(t_k) \approx \beta S_i(t_k)$ for all t_k and an appropriate $\beta > 0$. To prove some properties of this function, we simplify the notation by setting

$$a_k := S_j(t_k), \text{ and } b_k := S_i(t_k).$$

We also set $\boldsymbol{a} = \boldsymbol{S}_j = (S_j(t_1), ..., S_j(t_K))$ to denote the series of measurements and $\bar{a} = \bar{S}_j = \langle S_j(t_k) \rangle_{k=1}^K$ to denote its mean.

Assuming that a_k and b_k are non-negative, non-constant (i.e. there exist $a_\ell \neq a_k$ and $b_j \neq b_i$), and there exists a k such that $a_k \cdot b_k > 0$, we prove the following claims:

1. The infimum in (S5) is a minimum, i.e., there exists a $\beta > 0$, which minimizes the function

$$x \mapsto f_x(\boldsymbol{a}, \boldsymbol{b}) = \frac{2 \cdot \sum_{k=1}^K (a_k - x b_k)^2}{\sum_{k=1}^K (a_k - \bar{a})^2 + x^2 \sum_{k=1}^K \left(b_k - \bar{b}\right)^2}$$

2. The dissimilarity function is symmetric $d(\boldsymbol{a}, \boldsymbol{b}) = d(\boldsymbol{b}, \boldsymbol{a})$.

3. The dissimilarity function is invariant under scaling: $d(\boldsymbol{a}, c\boldsymbol{b}) = d(\boldsymbol{a}, \boldsymbol{b})$ for $c > 0$.

Ad 1. We discuss the mapping (1). First note that

$$\begin{aligned} \frac{\partial}{\partial_x} f_x(\boldsymbol{a}, \boldsymbol{b}) = - &\left[\sum_{k=1}^K (a_k - \bar{a})^2 + x^2 \sum_{k=1}^K \left(b_k - \bar{b}\right)^2 \right]^{-2} \times \\ &\left\{ 4 \cdot \sum_{k=1}^K b_k (a_k - x b_k) \left[\sum_{k=1}^K (a_k - \bar{a})^2 + x^2 \sum_{k=1}^K \left(b_k - \bar{b}\right)^2 \right] \right. \\ &\left. + 2 \cdot \sum_{k=1}^K (a_k - x b_k)^2 \left[2x \sum_{k=1}^K \left(b_k - \bar{b}\right)^2 \right] \right\} \end{aligned} \tag{S7}$$

That is, for $x \to 0$,

$$\frac{\partial}{\partial_x} f_x(\boldsymbol{a}, \boldsymbol{b}) \to - \left[\sum_{k=1}^K (a_k - \bar{a})^2 \right]^{-2} \times 4 \cdot \sum_{k=1}^K b_k a_k \left[\sum_{k=1}^K (a_k - \bar{a})^2 \right] < 0$$

Thus, (S5) is not minimal at x = 0. Considering the limit $x \to \infty$ we find that

$$\frac{\partial}{\partial_x} f_x(\boldsymbol{a}, \boldsymbol{b}) \to 0.$$

Further the numerator [second factor in (S7)] is negative for sufficiently large x, asymptoting to (note that cubic terms in the cancel)

$$-\sum_{k=1}^{K} \left[4a_k b_k \sum_{k=1}^{K} \left(b_k - \bar{b} \right)^2 \right] x^2$$

Thus, (S7) is strictly positive for sufficiently large x and an infimum at $x \to \infty$ cannot occur at ∞. By continuity, (1) must attain its minimum at a finite value of x.

□

Ad 2. Straightforward algebra shows that

$$f_x(\boldsymbol{a}, \boldsymbol{b}) = f_{1/x}(\boldsymbol{b}, \boldsymbol{a}).$$

Thus, if $x = x^*$ minimizes $x \mapsto f_x(\boldsymbol{a}, \boldsymbol{b})$, then $y^* = 1/x^*$ minimizes $y \mapsto f_y(\boldsymbol{b}, \boldsymbol{a})$ yielding the same minimum value.

□

Ad 3. Noting that

$$f_x(\boldsymbol{a}, c\boldsymbol{b}) = f_{cx}(\boldsymbol{a}, \boldsymbol{b}),$$

it is clear that if $x = x^*$ minimizes $x \mapsto f_x(\boldsymbol{a}, c\boldsymbol{b})$, $y^* = cx^*$ minimizes $y \mapsto f_y(\boldsymbol{a}, \boldsymbol{b})$ yielding the same minimum value.

□

References

[1] Robert E Buchanan. Life phases in a bacterial culture. *The Journal of Infectious Diseases*, pages 109–125, 1918.

[2] Karin Kovárová-Kovar and Thomas Egli. Growth Kinetics of Suspended Microbial Cells: From Single-Substrate-Controlled Growth to Mixed-Substrate Kinetics. *Microbiol. Mol. Biol. Rev.*, 62(3), 1998.

[3] Ursula Wanner and Thomas Egli. Dynamics of microbial growth and cell composition in batch culture. *FEMS Microbiol. Lett.*, 75(1):19–43, 1990.

[4] Daniel Wünsch, Kathleen Trautwein, Sabine Scheve, Christina Hinrichs, Christoph Feenders, Bernd Blasius, Dietmar Schomburg, and Ralf Rabus. Amino acid and sugar catabolism in the marine bacterium phaeobacter inhibens dsm 17395 from an energetic viewpoint. *Appl. Environ. Microbiol.*, 85, 2019.
